# Altered heart rate regulation by the autonomic nervous system in mice lacking natriuretic peptide receptor C (NPR-C)

**DOI:** 10.1038/s41598-017-17690-7

**Published:** 2017-12-14

**Authors:** Motahareh Moghtadaei, Ellen Langille, Sara A. Rafferty, Oleg Bogachev, Robert A. Rose

**Affiliations:** 1 Department of Physiology and Biophysics, Dalhousie University, Halifax, Nova Scotia Canada; 2 School of Biomedical Engineering, Faculty of Medicine, Dalhousie University, Halifax, Nova Scotia Canada; 3 Libin Cardiovascular Institute of Alberta, Cumming School of Medicine, University of Calgary, Calgary, Alberta Canada

## Abstract

Natriuretic peptides (NPs) play essential roles in the regulation of cardiovascular function. NP effects are mediated by receptors known as NPR-A, NPR-B or NPR-C. NPs have potent effects on regulation of heart rate (HR) by the autonomic nervous system (ANS), but the role of NPR-C in these effects has not been investigated. Accordingly, we have used telemetric ECG recordings in awake, freely moving wildtype and NPR-C knockout (NPR-C^−/−^) mice and performed heart rate variability (HRV) analysis to assess alterations in sympatho-vagal balance on the heart following loss of NPR-C. Our novel data demonstrate that NPR-C^−/−^ mice are characterized by elevations in HR, reductions in circadian changes in HR and enhanced occurrence of sinus pauses, indicating increased arrhythmogenesis and a loss of HRV. Time domain and frequency domain analyses further demonstrate that HRV is reduced in NPR-C^−/−^ mice in association with a reduction in parasympathetic activity. Importantly, the low frequency to high frequency ratio was increased in NPR-C^−/−^ mice indicating that sympathetic activity is also enhanced. These changes in autonomic regulation were confirmed using atropine and propranolol to antagonize the ANS. These findings illustrate that loss of NPR-C reduces HRV due to perturbations in the regulation of the heart by the ANS.

## Introduction

Natriuretic peptides (NPs), including atrial (ANP), B-type (BNP) and C-type (CNP) NPs, are a well-known group of peptide hormones that play essential and critical roles in the regulation of cardiovascular function in normal and disease conditions^1–3^. NPs are used extensively as biomarkers for cardiovascular disease and novel, synthetic NPs are in use and in development for therapeutic use in heart disease patients^1,4–6^. Nevertheless, our understanding of how NPs regulate cardiovascular function remains incomplete.

NPs exert their effects by binding to specific cell surface NP receptors (NPRs), which include NPR-A, NPR-B and NPR-C. All NPRs are highly expressed in the mammalian heart as well as in other tissues such as the brain and the nervous system^1,3,7^. Among the NPRs, NPR-C is the most widely and abundantly expressed NP receptor and it can bind to all NPs with comparable affinity^3,8,9^. NPR-A and NPR-B are coupled to the activation of guanylyl cyclases and cGMP signaling^10^. NPR-C, on the other hand, is functionally coupled to inhibitory G-proteins (G_i_), which inhibit adenylyl cyclases and activate phospholipid signaling upon NP binding^2,3,11^.

Heart rate (HR), a critical indicator of cardiac performance, is determined by the intrinsic rate of spontaneous activity in the sinoatrial node (SAN) and is importantly modulated by the autonomic nervous system (ANS)^12,13^. Specifically, the sympathetic nervous system (SNS) increases HR by increasing spontaneous action potential firing in SAN myocytes while the parasympathetic nervous system (PNS) decreases HR by reducing the frequency of spontaneous SAN action potential firing^14^.

We have shown that NPs have robust, potent effects on HR by modulating the electrophysiological properties of the SAN and have provided the first clear evidence that NPR-C can mediate some of these effects^2,7,15,16^. Specifically, we have shown that in the presence of acute β-adrenergic receptor (β-AR) activation, NPs reduce HR and slow electrical conduction in the SAN by inhibiting spontaneous action potential firing in SAN myocytes^16–18^. We have demonstrated that these inhibitory effects are mimicked by selective NPR-C agonists and that these effects are absent in NPR-C^−/−^ mice^17,18^, which shows that NPR-C is an important mediator of electrophysiological effects of NPs in the heart^19^. We have also shown that loss of NPR-C results in SAN dysfunction, as well as atrial arrhythmias, due to structural remodeling and fibrosis in the SAN and atria of NPR-C^−/−^ mice^18^.

Collectively, this prior work clearly illustrates an essential role for NPR-C in regulating HR and SAN function by mediating intrinsic effects of NPs within the heart. In contrast, much less is known about how NPR-C affects the regulation of HR by the ANS. A key role for the ANS is to produce fluctuations in HR, referred to as HR variability (HRV)^20,21^. This variability is a measure of the vital complexity in a healthy cardiovascular system and is responsible for the adaptability and functionality of this system. An absence or reduction in HRV compromises this normal complexity and is associated with pathological conditions in which the heart loses its adaptability^22^. Abnormalities in ANS inputs to the heart are known to be related to increased susceptibility to cardiovascular disease^23^. HRV is determined, at least in part, by the dynamic interactions between the sympathetic and parasympathetic nervous systems^24–26^ and, as such, HRV analysis enables the assessment of changes in the pattern of autonomic regulation of the heart that affect sympatho-vagal balance in the cardiovascular system^24,27,28^.

NPs have been found to contribute importantly to autonomic neurotransmission to the heart, and hence can have important effects on HRV^29–31^. These recent studies, which demonstrate that NPs, including BNP and CNP, have potent inhibitory effects on sympathetic neurotransmission and facilitate vagal neurotransmission, have directly implicated NPR-A and NPR-B in mediating these effects. Conversely, the role of NPR-C in these processes has not been investigated. Accordingly, the goal of the present study was to evaluate the role of NPR-C in ANS (SNS and PNS) regulation of the heart using telemetry recordings in conscious wildtype and NPR-C^−/−^ mice and performing HRV analysis on these recordings. Our novel data demonstrate that NPR-C^−/−^ mice are characterized by increased occurrence of arrhythmias in association with significantly lower variability in HR compared to wildtype mice.

## Materials and Methods

### Animals

This study utilized male littermate wild-type (NPR-C^+/+^) and NPR-C knockout (NPR-C^−/−^) mice between the ages of 10 and 15 weeks. NPR-C^−/−^ mice were originally from the Jackson Laboratory (strain B6;C-Npr3lgj/J). These mice were bred locally and backcrossed into the C57Bl/6 line for more than 15 generations. The NPR-C^−/−^ mouse contains a 36 base pair deletion that results in a truncated, non-functional NPR-C protein^19^. All experimental procedures utilized in this study followed the Canadian Council on Animal Care guidelines and were approved by the Dalhousie University Committee for Laboratory Animals.

### Telemetry ECG recording

ECGs and activity were monitored in awake, freely moving mice using telemetric recordings. To conduct these studies, mice were anesthetized (isoflurane inhalation) and telemetric ECG transmitters (HD-X11, Data Sciences International) were implanted subcutaneously. Wires were positioned around the heart in a lead II conformation. Mice were housed individually and each cage was placed on a receiver plate for data acquisition. After 7 days of recovery following transmitter insertion, telemetric ECG recordings and activity were acquired continuously for 48 hours. After this 48 hr recording period was completed, the effects of ANS blockade were investigated by intraperitoneal injection of the β-adrenergic receptor (β-AR) antagonist propranolol hydrochloride (10 mg/kg) or the muscarinic (M_2_) receptor antagonist atropine sulfate (10 mg/kg), with a minimum of 24 hrs between injections. Drug injections were performed at approximately the same time each day (9:00–11:00 AM). After injection, the ECG was recorded continuously for 1 hr and HRV analysis was performed on these recordings. All studies were performed in a dedicated telemetry room ensuring a quiet and undisturbed environment for the mice used in these experiments. ECG data acquisition, ECG filtering and R-wave detection was done using Ponemah software (Data Sciences International). HRV data analysis was performed using a custom Matlab script (see below). Activity was measured from telemeters as the relative movement of the animal, which is dependent on orientation and distance between the transmitter and receiver and quantified in counts per minute.

### Analysis of ECG recordings and heart rate variability

12 hr segments of light/dark (day/night) phases were selected from the complete recording period and were analyzed separately. For R-wave detection, each 12 hr segment was divided into six 2 hr segments. It is essential that HRV analysis be performed on sinus beats only; therefore, we initially scanned raw tachograms to identify sinus pauses and ectopic activity^24^. Ectopic beats, sinus pauses, obvious atrial and ventricular arrhythmias, and artifacts were invalidated and removed from the signal at this stage in order to ensure they were not included in our HRV analysis^24^. The effects of light/dark cycles on HRV were investigated in order to assess circadian changes by assessing global measures of cardiac function including mean RR interval, HR and HR (RR interval) histograms. For more detailed analysis of HRV, the low/high-activity phases were separated within light/dark segments based on activity measured from telemeters. This is important because even subtle changes in activity during the light or dark phases could occur and impact HRV. To separate low and high activity phases, activity (as measured from the telemeters) was quantified (counts/min) from locomotor activity plots. From these locomotor activity plots regions of minimal activity are easily identified and separated from periods of higher activity. We used an averaged activity of 2 counts/min as a cutoff to separate high and low activity whereby low activity was defined as an average activity level less than 2 counts/min in a segment of interest, while high activity was defined as an average activity level greater than 2 counts/min in a segment of interest. In our study, average activity levels were 0.5 ± 0.3 counts/min in low activity segments and 10 ± 1.5 counts/min in high activity segments. Parameters obtained from phases of high versus low activity during light or dark periods were then compared between wildtype and NPR-C^−/−^ mice.

### Time domain analysis

For time domain analysis, each of the day/night, low/high-activity phases were divided into 10 min episodes. Each episode was examined to ensure a stationary and stable sinus rhythm with no trend or periodic fluctuations. Next, R-wave detection was performed and RR interval time series were obtained. The time domain parameters we are reporting include: the standard deviation of all normal RR intervals (SDNN, in ms); the root mean square differences between successive RR intervals (RMSSD, in ms); and the percentage of normal consecutive RR intervals differing by >x ms (pNNx,%, in the present study x = 6 ms). SDNN is an index of total HRV that is best assessed from long term recordings, while RMSSD and pNNx are both beat to beat indexes of HRV, reflecting cardiac parasympathetic activity, which can also be assessed on short-term recordings^14,24^. Using a value of 6 ms for pNNx is reflective of relatively abrupt changes in HR^24^.

### Heart rate correction

A number of studies have reported that HRV is importantly impacted by HR itself due to the existence of an inverse exponential-like decay relationship between HR and HRV^32–34^. Recently, Monfredi *et al*.^32^ described an approach to correct for HR when performing HRV analysis. Using this approach, we plotted SDNN as function of HR for all wildtype and NPR-C^−/−^ mice in different conditions (low activity, high activity, after injection of propranolol, after injection of atropine) and fit these data with an exponential function, which was then used to generate the following equation to correct for HR and produce corrected SDNN (cSDNN) data.

$$cSDNN=\frac{SDNN}{{e}^{-0.0017\times HR}}$$

### Frequency domain analysis

For frequency domain analysis, each of the day/night, low/high-activity phases were divided into 2 min episodes. These time frames were chosen in order to ensure that each episode contained at least 1024 data points (R waves). Similar to the time domain analysis, each episode was manually examined to ensure a stationary and stable sinus rhythm, which is required for performing fast Fourier transforms (see below). Next, R-wave detection was performed and the RR interval time series were generated. Linear trends and drift were removed from the signal to reveal the HRV in the data. In the present study, we have used Welch’s method to characterize the frequency content of the signal, i.e. to estimate the power of the signal at different frequencies^35^. In Welch’s method, the signal is broken into overlapping segments to reduce noise in the frequency spectrum. Then, the segments are windowed to reduce spectral leakage. The periodogram of each windowed segment is calculated using the Fourier transform. Finally, the periodograms are averaged to make a single frequency spectrum. In the present study, we have used 50% overlapping and the Hamming window for the spectral density estimation^36^.

The total power of each periodogram was measured as a total index of HRV, which determines the integral of total variability over the entire frequency range. Then, the high frequency (HF) and low frequency (LF) components were extracted. The HF component of HRV (1.5–5 Hz) is predominantly mediated by the phasic activity of the parasympathetic nervous system^24^. The LF oscillations of HR (0.1–1.5 Hz) are regulated by both the sympathetic and parasympathetic nervous systems; however, the tonic sympathetic component is dominant^24^. We also quantified the LF/HF ratio, which is an indicator of sympatho-vagal balance^37^. We adopted these frequency ranges based on the location of the peaks formed in the HRV spectra of our data. The frequency ranges used in mice are distinct from those used in humans due to the differences in heart rate between the two species. The ranges we have utilized in our study are consistent with prior studies of HRV in mice^23,24^.

### Blood pressure recording

Blood pressure was measured in conscious restrained mice using a tail-cuff apparatus (IITC Life Sci) as we have reported previously^19^.

### Statistical analysis

All data are presented as means ± SEM. Data were analyzed using Student’s *t*-test, two-way ANOVA with a Holm-Sidak posthoc test or two-way repeated measures ANOVA with a Holm-Sidak posthoc test as indicated in each figure and table legend. *P* < 0.05 was considered significant.

### Data availability statement

The datasets generated during and/or analysed during the current study are available from the corresponding author on reasonable request.

## Results

**Figure 1:**
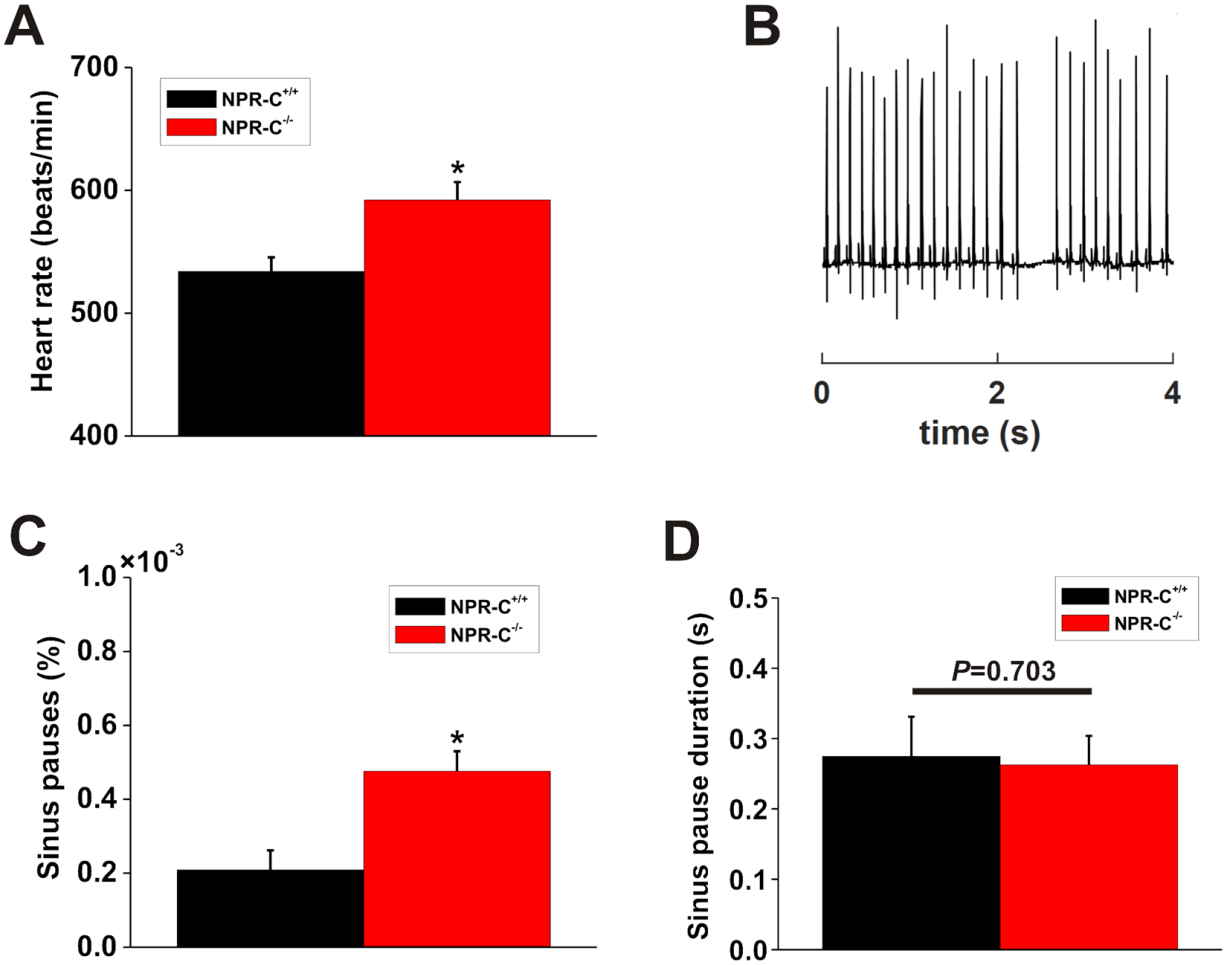
Incidence of sinus pauses in wildtype and NPR-C^−/−^ mice. (**A**) Summary of differences in HR in conscious, unrestrained NPR-C^+/+^ and NPR-C^−/−^ mice. (**B**) Representative telemetric ECG recording illustrating the occurrence of sinus pauses. (**C**) Summary data demonstrating the occurrence of sinus pauses in NPR-C^+/+^ and NPR-C^−/−^ mice. (**D**) Summary data demonstrating the duration of sinus pauses in NPR-C^+/+^ and NPR-C^−/−^ mice. *P < 0.05 vs NPR-C^+/+^ by Student’s *t*-test; *n* = 10 NPR-C^+/+^ and 10 NPR-C^−/−^ mice.

Initially we examined telemetric ECG recordings in wildtype and NPR-C^−/−^ mice for evidence of arrhythmias. HR, averaged over 48 hrs of recording, was elevated (*P* = 0.002; Fig. 1A) in NPR-C^−/−^ mice (592 ± 64 beats/min) compared to wildtype controls (534 ± 66.5 beats/min). Furthermore, we found that the incidence of sinus pauses was increased (*P* < 0.05) in NPR-C^−/−^ mice (Fig. 1B,C), consistent with an essential role for NPR-C in regulating SAN function. There was no difference in the duration of sinus pauses (*P* = 0.703) between wildtype and NPR-C^−/−^ mice (Fig. 1D). This increase in arrhythmogenesis occurred in the absence of any differences in blood pressure between wildtype and NPR-C^−/−^ mice as assessed acutely by tail-cuff plethysmography (Table 1).

**Table 1:** Blood pressure in NPR-C^+/+^ and NPR-C^−/−^ mice.

	NPR-C^+/+^	NPR-C^−/−^	P value
Systolic pressure (mmHg)	112.6 ± 21.1	114.7 ± 15	0.806
Diastolic pressure (mmHg)	80.2 ± 14.5	78.8 ± 13.9	0.825
Mean arterial pressure (mmHg)	90.5 ± 16.1	90.6 ± 13	0.994

Data analyzed by Student’s *t*-test; *n* = 10 NPR-C^+/+^ and 10 NPR-C^−/−^ mice.

**Figure 2:**
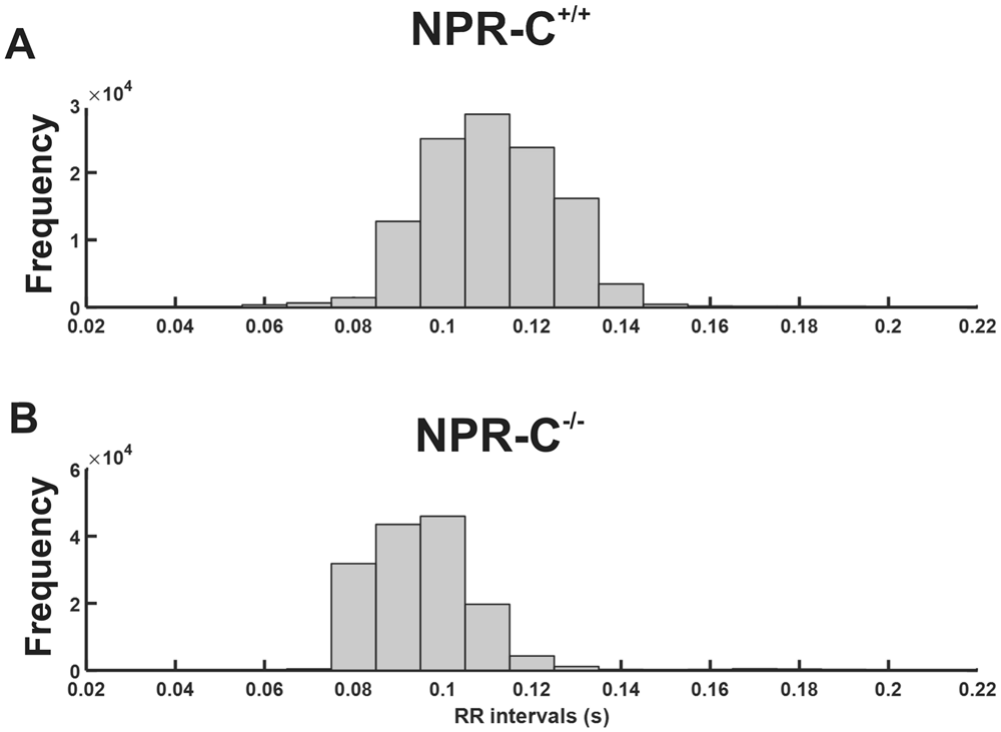
Variability in RR interval in wildtype and NPR-C^−/−^ mice. Representative RR-interval histograms generated from 24 hrs of telemetric ECG recording for NPR-C^+/+^ (**A**) and NPR-C^−/−^ (**B**) mice respectively. Bin width is 10 ms. These data, which demonstrate a reduction in HR variability in NPR-C^−/−^ mice, are representative of measurements made in 10 NPR-C^+/+^ and 10 NPR-C^−/−^ mice.

**Figure 3:**
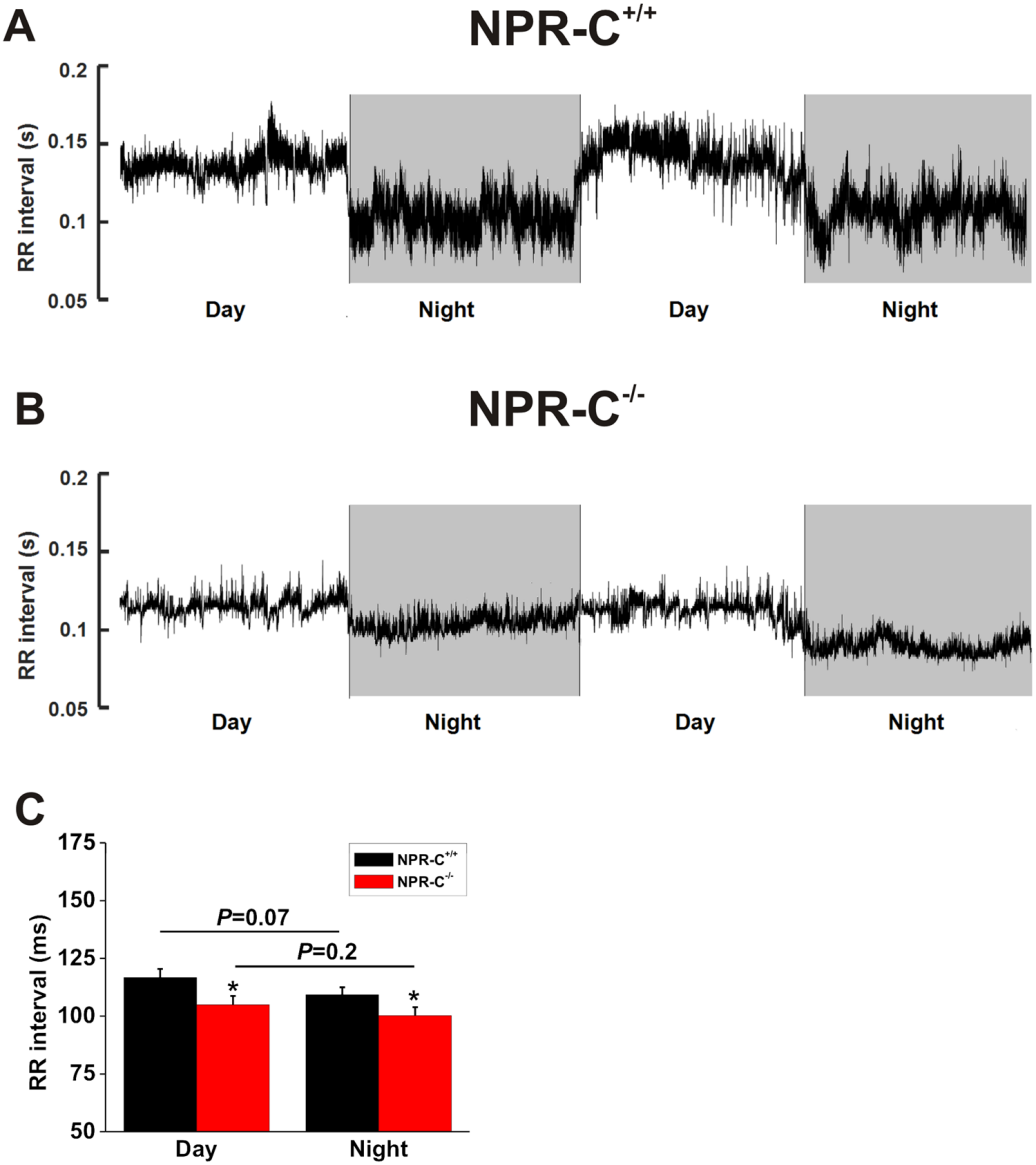
Circadian changes in heart rate in wildtype and NPR-C^−/−^ mice. Representative RR interval tachograms from 48 hrs of telemetric ECG recording in NPR-C^+/+^ (**A**) and NPR-C^−/−^ (**B**) mice. (**C**) Summary data illustrating mean RR interval in NPR-C^+/+^ and NPR-C^−/−^ mice during day and night cycles. *P < 0.05 using two-way ANOVA with a Holm-Sidak test; *n* = 10 NPR-C^+/+^ mice and 10 NPR-C^−/−^ mice.

Based on the above findings, we next investigated how RR intervals changed in different conditions in wildtype and NPR-C^−/−^ mice. An initial overview of the variability in HR in wildtype and NPR-C^−/−^ mice is illustrated in representative RR interval histograms obtained over 24 hrs (Fig. 2). These histograms demonstrate that the overall variability in RR interval (i.e. HR), as indicated by the frequency of occurrence of different RR interval bins, is reduced in NPR-C^−/−^ mice compared to wildtype controls. Furthermore, RR intervals where more frequently lower (i.e. HR was higher) in NPR-C^−/−^ mice (Fig. 2). Examination of circadian changes in RR interval during day and night phases further demonstrates that RR interval tended to be lower (i.e. HR tended to be higher; *P* = 0.07) at night in wildtype mice (Fig. 3A), as expected^24^. These day/night differences in RR interval were less pronounced in NPR-C^−/−^ mice (Fig. 3B) where, overall, there were no differences (*P* = 0.20) in RR interval between day and night phases (Fig. 3C). Summary data (Fig. 3C) also demonstrate that RR interval in NPR-C^−/−^ mice was lower (*P* < 0.05) than NPR-C^+/+^ mice during both day and night phases.

**Figure 4:**
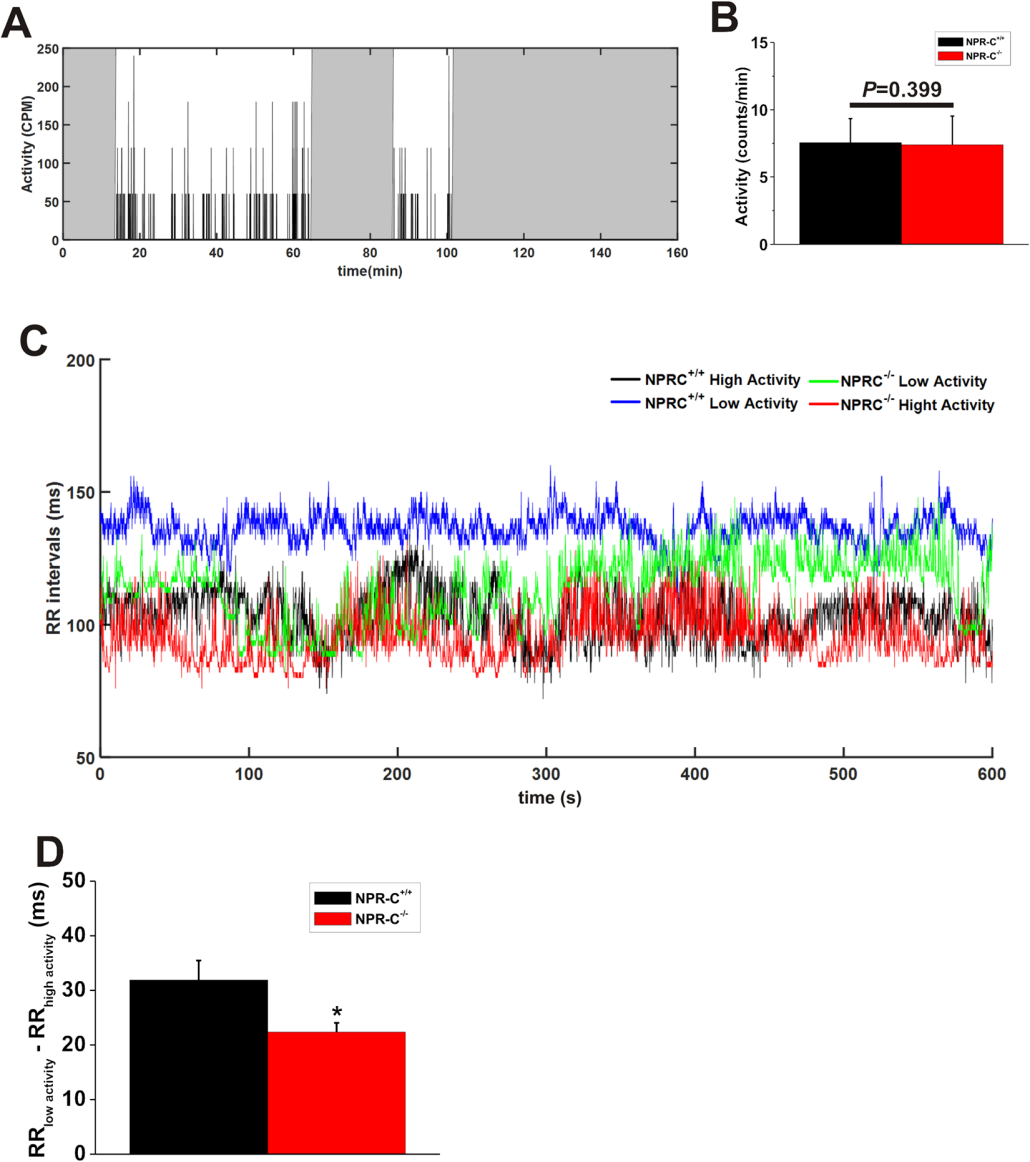
Differences in heart rate between wildtype and NPR-C^−/−^ mice during high activity and low activity phases. (**A**) Representative plot illustrating locomotor activity signals measured in counts per minute (cpm). The gray regions are classified as low activity phases and the white regions are considered as high activity phases. (**B**) Summary of activity levels (averages during low and high activity phases) between wildtype and NPR-C^−/−^ mice. Data analyzed using Student’s *t*-test. (**C**) Representative tachograms derived from 10 min sections of telemetric ECG recordings during low and high activity phases in NPR-C^+/+^ and NPR-C^−/−^ mice. (**D**) Summary data illustrating the difference between RR interval during low and high activity phases in NPR-C^+/+^ and NPR-C^−/−^ mice. *P < 0.05 by Student’s *t*-test; *n* = 10 NPR-C^+/+^ mice and 10 NPR-C^−/−^ mice.

To more accurately examine differences in RR interval during different physiological states, we identified periods of high activity and low activity (based on telemetrically recorded activity levels; Fig. 4A) in wildtype and NPR-C^−/−^ mice and assessed RR intervals using 10 min segments of ECG recordings. There were no obvious differences in activity patterns between wildtype and NPR-C^−/−^ mice from locomotion plots. Consistent with this, average activity (during low and high activity phases) was not different (*P* = 0.399) between genotypes (Fig. 4B). Representative tachograms (Fig. 4C) illustrate that wildtype mice have clear differences in RR interval between high activity and low activity phases. In contrast, NPR-C^−/−^ mice have shorter RR intervals and less separation in RR interval between high activity and low activity phases. Summary data show that the difference between RR interval during high activity and low activity phases is reduced (*P* < 0.05) in NPR-C^−/−^ mice compared to wildtypes (Fig. 4D). Collectively, the findings presented thus far demonstrate that conscious, freely moving NPR-C^−/−^ mice have elevations in HR during high activity and low activity phases, reductions in HRV and a reduced capacity to modulate HR in accordance with their level of activity in different physiological conditions.

**Figure 5:**
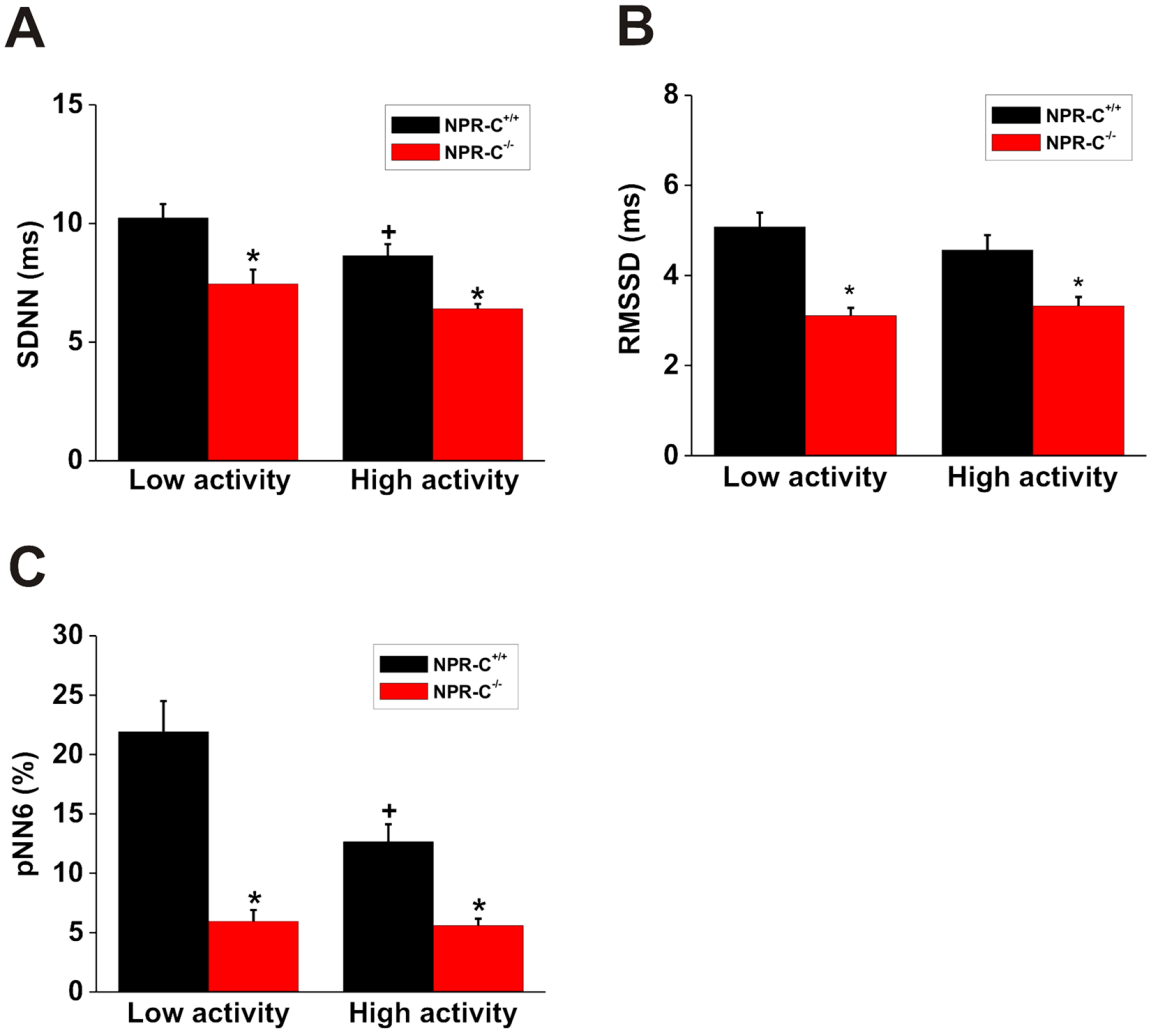
Assessment of HRV by time domain analysis in wildtype and NPR-C^−/−^ mice during low and high activity phases. Summary data illustrating (**A**) the standard deviation of all RR intervals (SDNN), (**B**) the root mean square of the difference of successive RR intervals (RMSSD) and (**C**) the percentage of normal consecutive RR intervals differing by >6 ms (pNN6) in NPR-C^+/+^ and NPR-C^−/−^ mice. *P < 0.05 vs. NPR-C^+/+^ within activity phase; ^+^ *P* < 0.05 vs low activity within genotype by two-way ANOVA with the Holm-Sidak posthoc test; *n* = 10 NPR-C^+/+^ mice and 10 NPR-C^−/−^ mice.

To investigate changes in HRV in more detail we next performed time domain analysis of HRV in wildtype and NPR-C^−/−^ mice (Fig. 5). Specifically, we measured SDNN, RMSSD and pNN6 during low activity and high activity phases (as assessed from telemetric activity measurements) in each genotype. SDNN (Fig. 5A), which is a measure of total HRV^24^, was reduced (*P* < 0.005) in NPR-C^−/−^ mice compared to wildtypes during low and high activity phases. Furthermore, SDNN was reduced during high activity in wildtype mice (*P* < 0.05), but not in NPR-C^−/−^ mice where there was only a trend towards reduction (*P* = 0.06). RMSSD (Fig. 5B) and pNN6 (Fig. 5C), which are both measures of PNS activity^24^, were each reduced (*P* < 0.005) in NPR-C^−/−^ mice during low and high activity phases. Similar to SDNN, pNN6 was also lower during the high activity phase in wildtype (*P* < 0.005), but not in NPR-C^−/−^ mice (*P* = 0.37).

**Figure 6:**
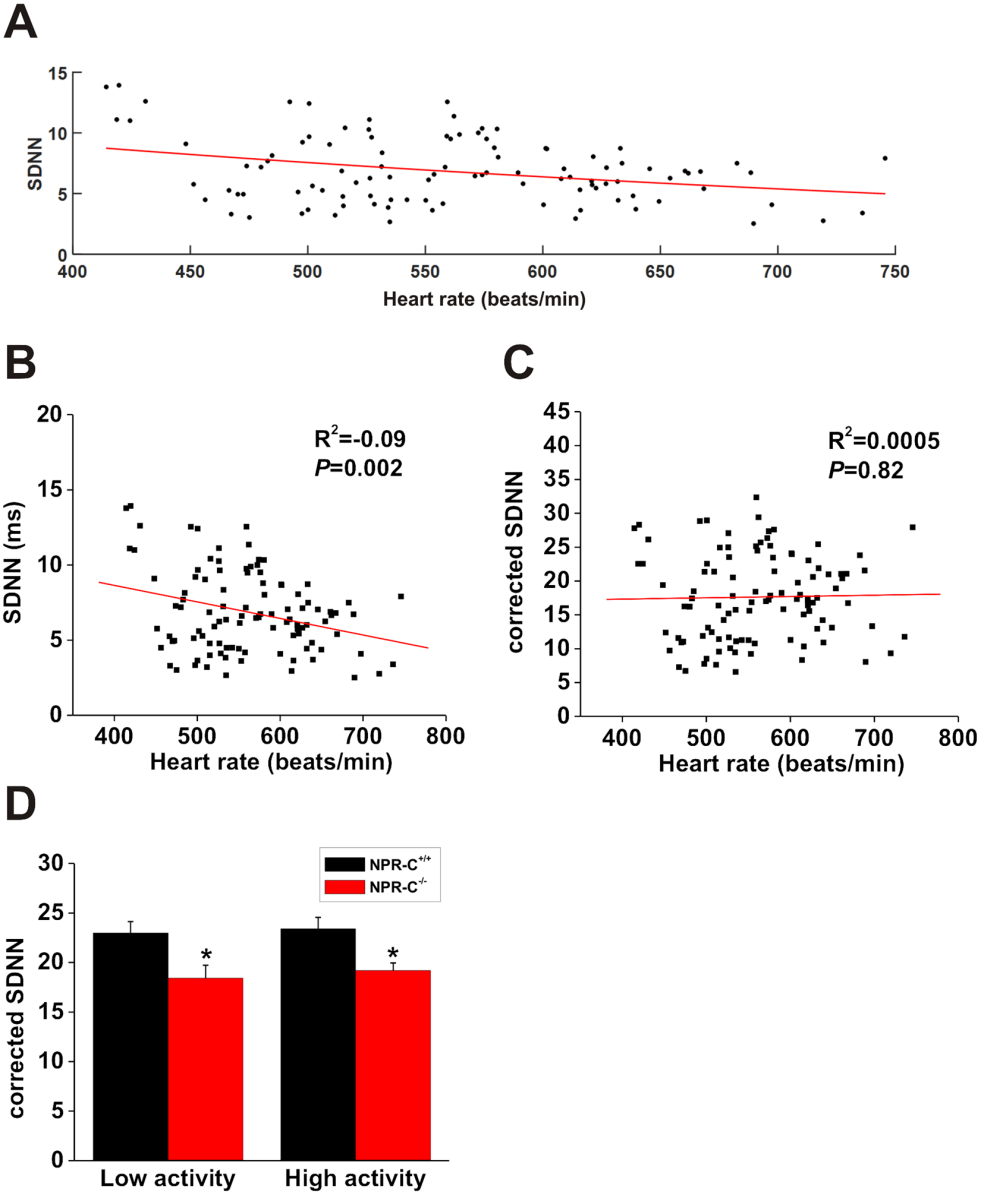
Assessment of HRV (SDNN) in wildtye and NPR-C^−/−^ mice after correcting for heart rate. (**A**) Plot of SDNN in NPR-C^+/+^ and NPR-C^−/−^ mice in different conditions as a function of HR. Data were fit with an exponential function to correct for HR as detailed in the methods. (**B**) Linear regression analysis of uncorrected SDNN as a function of HR in NPR-C^+/+^ and NPR-C^−/−^ mice (same mice as used in panel A). (**C**) Linear regression analysis of corrected SDNN as a function of HR in NPR-C^+/+^ and NPR-C^−/−^ mice. Correlation coefficients in panels B and C obtained using Pearson’s correlation. (**D**) Summary data illustrating corrected SDNN in NPR-C^+/+^ and NPR-C^−/−^ mice. *P < 0.05 vs. NPR-C^+/+^ within activity phase by two-way ANOVA with the Holm-Sidak posthoc test; *n* = 10 NPR-C^+/+^ mice and 10 NPR-C^−/−^ mice.

While the above analysis clearly indicates that HRV is reduced in NPR-C^−/−^ mice, it is important to recognize that HRV is affected by HR itself ^33,34^. Accordingly, we corrected HRV for HR using recently described mathematical approaches^32^ (see also methods). Specifically, we plotted SDNN as a function of HR from wildtype and NPR-C^−/−^ mice under a number of conditions (low activity, high activity, after injection of propranolol, after injection of atropine) to ensure a range of HRs were included (Fig. 6A). These data were fit with an exponential function and corrected for HR using the approach of Monfredi *et al*.^32^. Figure 6B and C illustrate the relationships between SDNN and HR before and after correction for HR. Linear regression analysis of uncorrected SDNN (same data as Fig. 6A) as a function of HR shows an R^2^ value of −0.09 (*P* = 0.002; Fig. 6B) indicating that HR accounts for ~10% of the variability before correction for HR. In contrast, linear regression analysis of corrected SDNN as a function of HR showed no correlation (R^2^ = 0.0005; *P* = 0.82; Fig. 6C) indicating that SDNN was successfully corrected for HR. Figure 6D illustrates that corrected SDNN remained reduced (*P* < 0.05) in NPR-C^−/−^ mice in low activity and high activity phases. In summary, time domain analyses demonstrate that total HRV was reduced in NPR-C^−/−^ mice in association with reductions in PNS activity, even after accounting for possible effects of HR itself.

**Figure 7:**
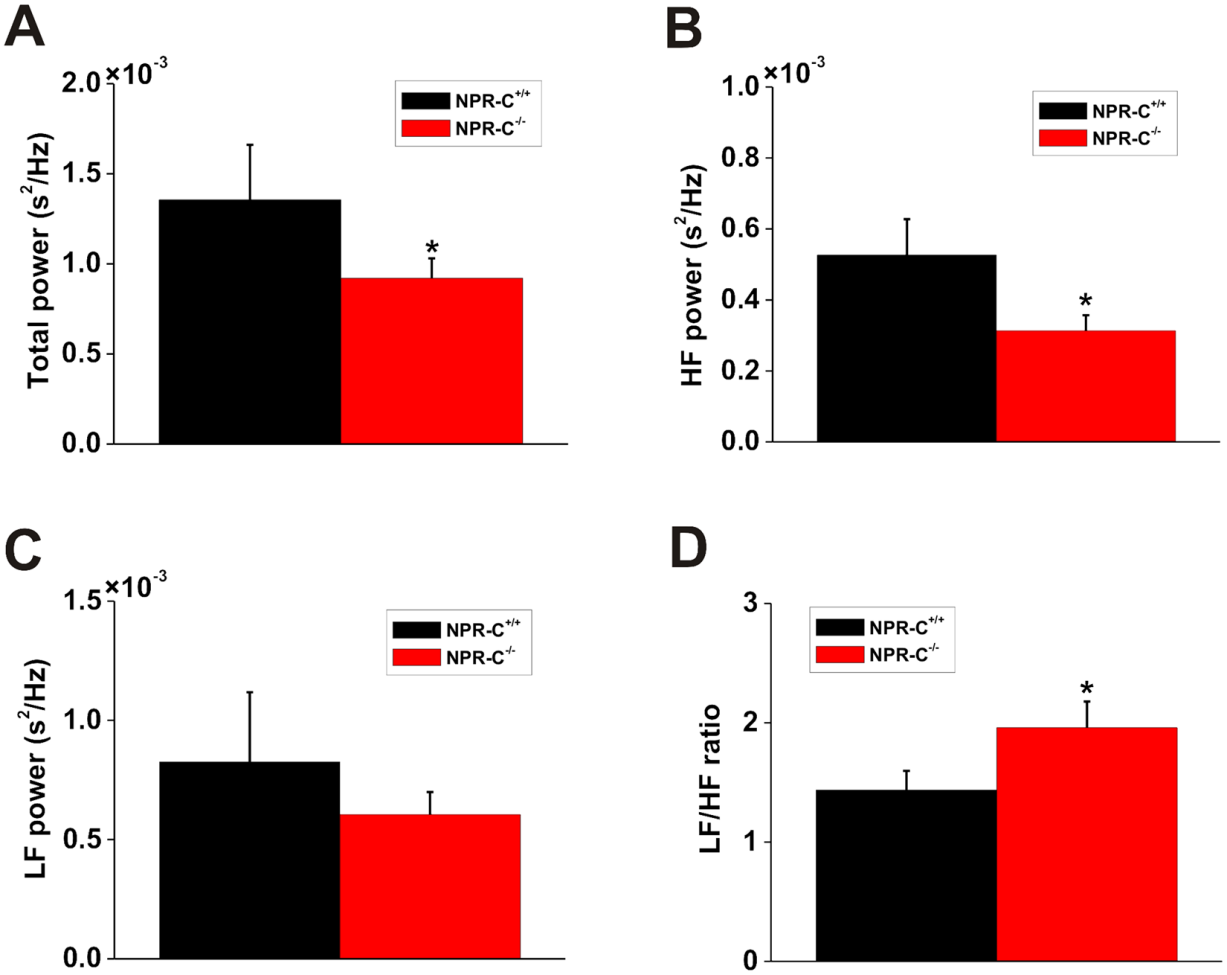
Assessment of HRV by frequency domain analysis in wildtype and NPR-C^−/−^ mice during high activity. Summary data illustrating total power of the HRV spectra (**A**), power in the HF band (**B**), power in the LF band (**C**), and the LF/HF ratio (**D**) in NPR-C^+/+^ and NPR-C^−/−^ mice. *P < 0.05 vs. NPR-C^+/+^ by Student’s *t*-test; *n* = 10 NPR-C^+/+^ mice and 10 NPR-C^−/−^ mice.

HRV in wildtype and NPR-C^−/−^ mice was also examined using frequency domain analysis (Fig. 7). Frequency domain analysis was performed only on data obtained during high activity (as assessed by telemetry) because activity levels were similar between genotypes and because our time domain analysis shows that changes in NPR-C^−/−^ mice were similar in low and high activity. Total power (Fig. 7A) was reduced (*P* < 0.05) in NPR-C^−/−^ mice compared to wildtype controls indicating an overall reduction in HRV. Similarly, power in the HF band (Fig. 7B), which is representative of PNS activity, was reduced (*P* < 0.05) in NPR-C^−/−^ mice. Conversely, power in the LF band (Fig. 7C), which is determined by both PNS and SNS activity^24^, was not different (*P* = 0.17) between wildtype and NPR-C^−/−^ mice. Finally, we measured the LF/HF ratio (Fig. 7D), which is a measure of sympatho-vagal balance^24^, in wildtype and NPR-C^−/−^ mice. These data show that the LF/HF ratio is increased (*P* < 0.05) in NPR-C^−/−^ mice indicating an increase in sympathetic activity. Collectively, frequency domain analysis demonstrates a loss of HRV in NPR-C^−/−^ mice in association with a shift in sympatho-vagal balance towards reduced PNS activity as well as increased SNS activity.

**Figure 8:**
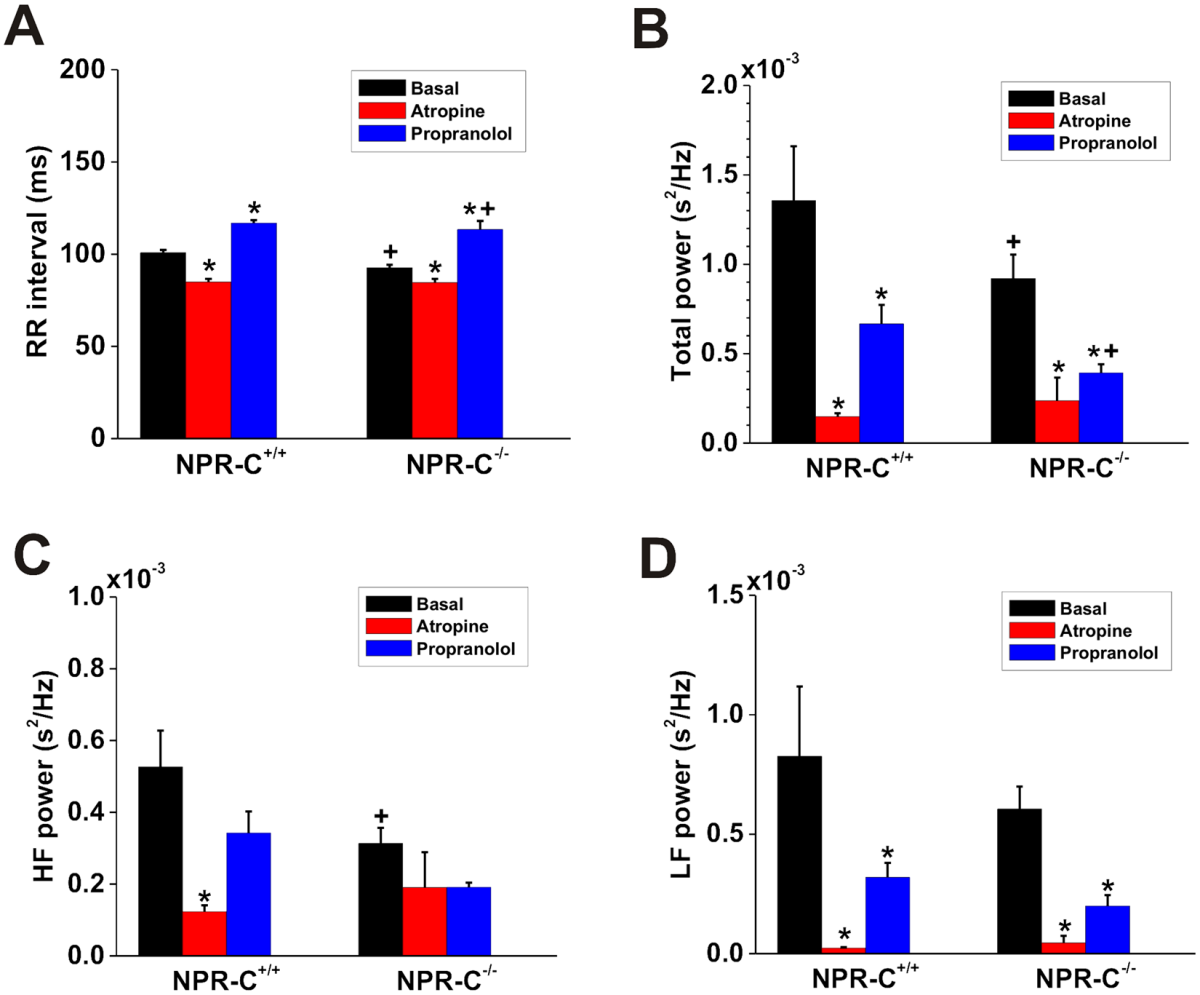
Effects of autonomic nervous system blockers on RR interval and frequency domain measures of HRV in wildtype and NPR-C^−/−^ mice. Summary data illustrating the effects of atropine or propranolol on RR interval (**A**), total power of the HRV spectra (**B**), power in the high frequency band (**C**) and power in the low frequency band (**D**) in NPR-C^+/+^ and NPR-C^−/−^ mice. *P < 0.05 vs. basal condition within genotype; ^+^ *P* < 0.05 vs. NPR-C^+/+^ within treatment condition by two-way repeated measures ANOVA with the Holm-Sidak posthoc test; *n* = 10 wildtype and 10 NPR-C^−/−^ mice.

To further investigate changes in autonomic balance in NPR-C^−/−^ mice we measured the effects of atropine and propranolol on RR interval (Fig. 8A) as well as on frequency domain measures of HRV (Fig. 8B–D). These experiments were performed on data acquired during high activity. As expected, atropine decreased (*P* < 0.05) RR interval (i.e. increased HR) and propranolol increased (*P* < 0.05) RR interval (i.e. decreased HR) in both genotypes (Fig. 8A). Importantly, however, the effects of atropine were smaller and the effects of propranolol were larger in NPR-C^−/−^ mice. Because of this differential response, RR interval was no longer different (*P* = 0.879) between genotypes after application of atropine (Fig. 8A). RR interval after application of propranolol remained lower (*P* < 0.05) in NPR-C^−/−^ mice (Fig. 8A). These results are consistent with a decrease in PNS activity as well as an increase in SNS activity in NPR-C^−/−^ mice.

Atropine decreased (*P* < 0.05) total power (Fig. 8B), HF power (Fig. 8C), and LF power (Fig. 8D) to similar levels in wildtype and NPR-C^−/−^ mice. Because total power and HF power are reduced (*P* < 0.05) in NPR-C^−/−^ mice at baseline the magnitude of the effect of atropine was smaller (consistent with less PNS activity) in NPR-C^−/−^ mice. Similarly, propranolol reduced (*P* < 0.05) total power in wildtype and NPR-C^−/−^ mice; however, the effects were larger in NPR-C^−/−^ mice, which, along with the baseline difference in total power between genotypes, resulted in lower (*P* < 0.05) total power in NPR-C^−/−^ mice after SNS blockade compared to wildtypes (Fig. 8B). Larger effects of propranolol in NPR-C^−/−^ mice are consistent with increased SNS activity in these mice. HF power was not affected by propranolol in wildtype (*P* = 0.607) or NPR-C^−/−^ (*P* = 0.613) mice, which is consistent with HF power being mainly determined by PNS activity (Fig. 8C). LF power, which is determined by both PNS and SNS activities, was reduced by propranolol (*P* < 0.05) in wildtype and NPR-C^−/−^ mice (Fig. 8D).

## Discussion

In the present study we have used telemetric ECG recordings in wildtype and NPR-C^−/−^ mice to study the role of NPR-C in autonomic regulation of the heart. Our long-term telemetric ECG monitoring allowed us to investigate how loss of NPR-C impacts cardiac rhythm in conscious, freely moving NPR-C^−/−^ mice, which has not been previously done. Strikingly, our data demonstrate that NPR-C^−/−^ mice are characterized by increased incidences of sinus pauses, elevated HR and an impaired capacity to modulate HR according to activity level in different physiological conditions such as at night vs. during the day.

In healthy mammals, including humans, HR is adjusted by the dynamic interactions between the sympathetic and parasympathetic nervous systems^24^, which is known as HRV. Analysis of HRV has commonly been used to evaluate changes in ANS regulation of the heart and to assess sympatho-vagal balance in the heart at the level of the SAN^23,24^. Specifically, regulation of the SAN by the parasympathetic nervous system is phasic and characterized by abrupt and fast changes in HR, while regulation by the sympathetic nervous system is tonic, occurring over a slower time scale^20^. Because we observed alterations in HR in NPR-C^−/−^ mice in association with loss of dynamic changes in HR in different physiological conditions (i.e. low vs. high activity), we assessed HRV in these mice using well-established approaches. Our measurements of HRV using both time domain and frequency domain analyses demonstrate that HRV is reduced in NPR-C^−/−^ mice as indicated by reductions in SDNN (time domain) and total power (frequency domain). This occurred in association with alterations in PNS mediated regulation of the heart in NPR-C^−/−^ mice as indicated by reductions in RMSSD and pNN6 (time domain measures of PNS regulation) as well as power in the high frequency band (frequency domain measure of PNS activity). The observation that both time domain (RMSSD) and frequency domain (HF power) measures of HRV, which are thought to be regulated by PNS activity^24^, were reduced in NPR-C^−/−^ mice strongly supports the conclusion that loss of NPR-C is associated with reduced parasympathetic activity.

In addition, our experiments also indicate that sympathetic activity is enhanced in NPR-C^−/−^ mice. Specifically, the LF/HF ratio, which is an indicator of sympatho-vagal balance^27,28^, was increased in NPR-C^−/−^ mice suggesting increased SNS activity. Although LF/HF ratio as a measure of sympatho-vagal balance has been questioned^38^, the conclusion that sympathetic activity is enhanced in NPR-C^−/−^ mice is further supported by experiments with the β-AR antagonist propranolol, which elicited larger increases in RR interval (i.e. reductions in HR) in NPR-C^−/−^ vs. wildtype mice. Total power was also reduced to a larger extent and hence was lower after application of propranolol in NPR-C^−/−^ mice. Collectively, our results demonstrate that HRV is reduced in NPR-C^−/−^ mice in association with reduced PNS activity as well as increased SNS activity. Our findings are also consistent with prior studies showing that propranolol prolongs the RR interval and decreases the LF component of HRV while atropine decreases the RR interval and decreases both the HF and LF component of the HRV in wildtype mice^33,39,40^. It should be noted that HRV may be affected by factors separate from the ANS and it is possible that HF and LF measures of HRV are not pure readouts of the two branches of the ANS^41,42^. Nevertheless, our conclusions that altered HRV in NPR-C^−/−^ mice is associated, at least in part, with changes in autonomic function are supported by our experiments using the ANS blockers atropine and propranolol, which elicited differential effects in NPR-C^−/−^ mice.

It has been reported that HRV depends importantly on HR itself whereby there exists an exponential decay relationship between HR and HRV^32,33,43,44^. This has created questions about the interpretation of studies of HRV^32^. Accordingly, we applied recently described mathematical approaches to correct SDNN measurements for HR^32^. From these analyses, our data show that even without correction HR accounted for less than 10% of the variability (R^2^ = −0.09 for SDNN vs. HR). This is not surprising as HR is relatively high in mice, falling in the range of the exponential decay curve where HR has much less of an impact on HRV^32^. Nevertheless, we were able to correct for this HR effect and demonstrate that corrected SDNN was still significantly reduced in NPR-C^−/−^ mice, clearly indicating that autonomic regulation of the heart is altered by loss of NPR-C. Our experiments with ANS blockers further support the conclusion that cardiac autonomic regulation is indeed perturbed in NPR-C^−/−^ mice.

It is well established that autonomic imbalance, characterized by a hyperactive SNS and a hypoactive PNS, is associated with a lack of dynamic flexibility and health, leading to pathological conditions including cardiovascular disease^45^. Also, it is well accepted that loss of HRV is associated with worse cardiovascular outcomes including hypertension, cardiac hypertrophy, heart failure and sudden death^24,46,47^. Prior studies have also shown that alterations in HRV are associated with SAN dysfunction (i.e. sick sinus syndrome), which is a major cause of sudden death in heart failure^24,48^. Our present work demonstrating more sinus pauses and elevated heart rate in NPR-C^−/−^ mice is in agreement with the concept that NPR-C plays a critical role in the regulation of the SAN by the autonomic nervous system and that loss of NPR-C is proarrhythmic due in part to a loss of HRV. Importantly, we found no differences in blood pressure, measured by tail-cuff plethysmography, between wildtype and NPR-C^−/−^ mice, suggesting that the alterations in autonomic regulation of the heart we have observed in NPR-C^−/−^ mice do not occur secondarily to any changes in blood pressure. It should be noted; however, that we did not obtain telemetric blood pressure data in our studies. As such, it is possible that there could be diurnal differences in blood pressure in NPR-C^−/−^ mice that were not detected in our study. We have also previously demonstrated that there are no changes in expression of NPR-A or NPR-B in our NPR-C knockout mice^7,19^; therefore, we conclude that the effects we have observed in NPR-C^−/−^ mice are directly related to the loss of NPR-C and are not associated with compensatory changes in other NPRs.

We have previously studied the effects NPR-C ablation on intrinsic SAN function and arrhythmogenesis^19^. This prior work demonstrates that SAN function is impaired in NPR-C^−/−^ mice (as indicated by a prolongation of SAN recovery time), which is consistent with our findings in the present study showing that conscious, unrestrained NPR-C^−/−^ mice demonstrate an increase in sinus pauses. Our previous work also showed that impaired intrinsic SAN function in NPR-C^−/−^ mice occurred in association with enhanced fibrosis in the SAN, which resulted in a slowing of SAN electrical conduction^19^. Thus, when considered collectively, our past and present work shows that loss of NPR-C results in impaired conduction in the SAN as well as alterations in sympatho-vagal balance. Each of these alterations is likely to contribute to the increase in arrhythmogenesis, including sinus pauses and SAN dysfunction, in NPR-C^−/−^ mice. While the ANS is an important determinant of HRV, it is now thought that changes in HRV can also arise from alterations in the intrinsic properties of pacemaker cells in the SAN and the responsiveness of these cells to external inputs in the absence of changes in ANS activity^24^. While our study demonstrates that changes in autonomic regulation contribute importantly to the loss of HRV in NPR-C^−/−^ mice we cannot exclude the possibility that changes in SAN myocyte properties are also a factor, especially given that we have previously identified intrinsic alterations in SAN function in NPR-C^−/−^ mice. We have previously performed complete ANS blockade in wildtype and NPR-C^−/−^ mice (a maneuver that allows for the measurement of intrinsic heart rate *in vivo*) and found that SAN function is indeed impaired in NPR-C^−/−^ mice^19^. This will be an important area for ongoing investigation that could be addressed by measuring the effects of direct stimulation of cervical parasympathetic nerves and stellate ganglia in NPR-C^−/−^ mice and by assessing variability in isolated SAN preparations, including isolated pacemaker cells, which lack neural regulation. While our experiments with atropine and propranolol do support the conclusion that NPR-C regulates heart rate and HRV via the ANS they raise the interesting possibility that some of these effects may involve differences in autonomic receptors and/or their downstream signaling pathways in SAN cells. Furthermore, it is conceivable that effects of NPR-C on autonomic neurotransmission could involve accentuated antagonism whereby altered effects on one branch of the ANS could affect how the other branch of the ANS responds^49,50^. These will also be important areas of future investigation. Such studies will further our understanding of the role of NPR-C in regulating HRV via the ANS as well as via mechanisms intrinsic to the SAN. Respiratory frequency can also alter heart rate and HRV independently of changes in autonomic regulation^21^, but whether this is altered in NPR-C^−/−^ mice is unknown. The role of NPR-C in regulating respiratory frequency and hence HRV could be another avenue for future study.

Many circulating humoral factors can affect heart rate and HRV and, as our understanding of these increases, it will be important to synthesize this information so that we can understand and interpret the relative importance of the many neurohumoral factors that could regulate these important physiological processes. NPs are of particular interest because they are synthesized locally in the atria of the heart, including in the vicinity of the SAN, and are known to have potent effects on heart rate and cardiac electrophysiology^2,3^. Our work clearly demonstrates that NPs play a vital role in the regulation of the heart by the autonomic nervous system and demonstrate for the first time that NPR-C is critically involved in these effects. Interestingly, several prior studies have also shown that NPs regulate autonomic control of the heart. For example, BNP and CNP have both been shown to facilitate vagal neurotransmission via a guanylyl cyclase-linked pathway involving NPR-A and NPR-B^29^. Similarly, it has recently been shown that CNP, acting via NPR-B, can reduce sympathetic neurotransmission by modulating norepinephrine release^31^. This latter conclusion is based on data showing that a neuron specific dominant negative NPR-B rat demonstrates elevated heart rate, reduced HRV and a shift to sympatho-excitation. Based on these prior findings as well as our present study on NPR-C, it will be important to continue to investigate the effects of different NPs and NPRs in these processes. In particular, it is essential to recognize that NPs can bind to more than one NPR simultaneously^3,18^; therefore, it is likely that the effects of NPs on autonomic neurotransmission and HRV will be determined by the relative levels of activation of these different NPRs in different physiological and pathophysiological conditions as well as in disease states where receptor expression may be altered.

In conclusion, we have demonstrated that loss of NPR-C is associated with increased HR, enhanced arrhythmogenesis and a reduced capacity to modulate heart rate in different physiological conditions in conscious, unrestrained mice in association with a loss of HRV and a shift in sympatho-vagal balance. Our work adds to a growing body of literature demonstrating that NPs play essential roles in the regulation of the heart by the autonomic nervous system and that perturbations in these processes are proarrhythmic. This indicates the NP system, and NPR-C in particular, could be a viable therapeutic target for optimizing autonomic neurotransmission in the heart.
